# Supplementary material for: Formation of polarity convergences underlying shoot outgrowths
Source: eLife. 2016 Aug 1;5:e18165. doi: 10.7554/eLife.18165 (PMC4969039; doi:10.7554/eLife.18165)
Supplement: Source code 3. — DOI: http://dx.doi.org/10.7554/eLife.18165.031 [file elife-18165-code3.zip › Source code 3_Fig. 7C/description.html]

  Mediated Polarisation Model  


# Mediated Polarisation Model

## Representation of the tissue

To start with, the tissue is represented using a cell complex, but with walls subdivided so they are represented not
with one, but many wall elements (Fig. 1). Each element can hold a different concentration of mediator or polariser
concentration.

|  |
| --- |
|  |
| Fig. 1 - Cell representation with interior representing mediator concentration and walls representing polarisers concentrations |

To be able to simulate the reactions involving chemicals in the membrane, the cell itself and the cell wall, the
tissue is also represented as a graph in which each element is represented as a vertex, and edges denote an
interface through which chemicals can diffuse or be transported (Fig. 2). There are three types of elements:

1. Cells (in red)
2. Membranes (in green)
3. Cell walls (in purple)

|  |
| --- |
|  |
| Fig. 2 - Representation of the tissue with the graph used to solve the equations |

## Chemical reactions

There are three chemical elements: the polarisers *h* (head) and *b* (base, or tail), and the mediator *m*.
The chemical reactions are different depending on the element considered.
Let's first consider the chemical reactions on the cell itself.

1. ### Chemical reactions in the membranes

   For this, we need the concentration of the chemicals in the cell, denoted *hc* or
   *bc* (there is no mediator free in the cell). We also need the amount of mediator in the cell wall
   *mw*, and the length of the wall, *lw*. At last, the diffusion exist only within
   the membrane or cell wall.

   In these equations, ρ denote either the default binding rate or the production rate, μ the unbinding or
   degradation rate, η the self-activation of the binding, α the influence of the mediator (in binding the
   head or unbinding the tail), ν denote the trans-membrane permeability, ψi the influx transport,
   ψo the efflux transport and D the diffusion within the membrane, and δm denotes
   the thickness of the membrane.
2. ### Chemical reactions in the cells

   The cells don't have mediator, as it's supposed to diffuse slowly along the membrane and not very fast within the
   cell. Therefore, we only need to account for the head and tail polarisers in the cell c: *hc* and
   *bc*. Also, the concentration will depend on the membranes surrounding the cell. The set of all
   the neighbors of the vertex representing the cell is denoted . The concentrations
   in the neighbor n are all indexed with n: *hn*, *bn* and *mn*.

   In these equations, in addition to the notations introduced previously,  denotes
   the area of the cell, and  denotes the area of a specific membrane (i.e. the
   length of the membrane time the equivalent thickness of it).
3. ### Chemical reactions in the cell walls

   In these equations, in addition to the notations already introduced, δw denotes the thickness of
   the cell wall and *Dw* the diffusion coefficient of the mediator within the cell wall.
