# Supplementary material for: Formation of polarity convergences underlying shoot outgrowths
Source: eLife. 2016 Aug 1;5:e18165. doi: 10.7554/eLife.18165 (PMC4969039; doi:10.7554/eLife.18165)
Supplement: Supplementary file 1. — Instructions on how to run models and explanation of the code for each model. DOI: http://dx.doi.org/10.7554/eLife.18165.051 [file elife-18165-supp1.docx]

For each of the three models used here (up-the-gradient, flux-based and indirect coupling), code is provided for each simulation used for the paper, named according to the Fig. number. In most cases there are only small differences to the code / parameters for each simulation. These differences are described below.

The model folders can be opened in L-studio with VVe (downloaded from <http://algorithmicbotany.org/virtual_laboratory/>) by dragging the model folder into the L-studio window. The most informative parts of the model are under the “Program” and “Parameters” tabs. “Program” contains the code used to initialise the system, specify the model and draw the system. “Parameters” contains the list of parameter values. To compile a model, click the VVE tab in L-studio and then click “build”. To run a model, click “VVE” then “run”.

For a detailed description of how models are implemented in VVE, see the Model descriptions of Abley et al., 2013. In brief, the tissue is represented by two graphs, a tissue graph (which contains cell nodes, membrane nodes and wall nodes (the latter only in the indirect coupling model) and a solver graph which contains only one type of node. Concentrations of chemicals are transferred between the two graphs at each step of the simulation. The system is initialised with chemicals in the tissue graph. The concentrations of chemicals are then transferred to the equivalent nodes in the solver graph. The solver graph is used to solve the equations for the models. Concentrations of chemicals are then transferred back to the tissue graph each step so the tissue can be drawn.

The most important parts of the program for the implementation of each model are the following functions, which can be found in the code using the Find tool:

**initConcentrations**

This initialises the system with specific concentrations of chemicals

**setCellNodeOrganiser**

This specifies the behaviour of the tissue boundaries

**updateDerivatives** (and the functions called from here)

Specifies the interactions between chemicals in different compartments so that derivatives of the model can be calculated by the solver.

**updateOrganisers**

Adds any extra regions of auxin production / removal or import that are used in the simulation (other than the regions at the tissue boundaries).

Details of differences between models.

**Up the gradient model**

**Fig. 8A** -basic model for small tissue

**Fig. 8B**

Same as Fig. 8A, but the GridSize parameters are changed in the parameter file to make the tissue larger. Also, so that the tissue is set up correctly, in the function TurnIntoSquares, line 1470, should read if (T.border(c) or (c->pos[1] > **15**) ). In the initConcentrations function, lines 1201 and 1207 modified to correctly specify positions of the ends of the tissue:

Line 1201: if (c->pos[0] > **22.5**) //setting high auxin concentration at distal end

Line 1207: else if (c->pos[0]**< 3**) //setting low auxin concentration at proximal end

In updateDerivatives function, line 2203, the conditional statement is altered so that the last part states nm->isBottomMembrane and nm->bottomMembraneNumber <**26.** The only difference is that 26 is used instead of 18 (just to account for tissue being larger)

**Fig 11H.**

Same as Fig. 8B, except initialisation is changed so that the distal end doesn’t start off with high auxin.

In initConcentrations, line 1200, the auxin concentration at the distal end is set to be 0.5 instead of 50:

if (c->pos[0] > 22.5) //setting auxin concentration at distal end

{

c->auxin = **0.5**;

Also, the setCellNodeOrganiser function is changed so that line 1253 onwards reads

if(type == C_MINUS) // setting distal end to have high import and degradation

{

n->import=importInMinus; //this is set to be 2 in parameter file

n->degradation =minusLevel[0]; // this is set to be 0.01 in parameter file

}

else if (type== C_PLUS) // setting proximal end to have high production

{

n->degradation =0.05; //the proximal end has elevated degradation, this is the same value as //in Fig 8B

}

break;

As mentioned in the comments above, in the parameter file, MinusLevel = 0.01 and ImportInMinus = 2.

To allow elevated auxin import to be induced when the intracellular auxin concentration exceeds a threshold level, an updateOrganisers function is added (line 1729) and called each step on line 1711. Parameters associated with this function are added to the parameter file (ThresholdToBecomeMinus: 8; TimeForMinus: 0).

**Fig. 14D**

As for Fig 8A, but in init concentrations, auxin in all cells is set to 0.5. Line 1200 onwards are:

if (c->pos[0] > 15) //setting high auxin concentration at distal end

{

c->auxin = 0.5;

c->PIN = 0;

c->type=C_MINUS;

}

else if (c->pos[0]< 3) //setting low auxin concentration at proximal end

{

c->auxin = 0.5;

c->PIN = 0;

c->type=C_PLUS;

}

else

{

c->auxin = 0.5;

c->PIN = 0;

c->type=C_NORMAL;

}

Also, in setCellNodeOrganiser function, the top of the tissue (minus org) is set to have elevated auxin import and degradation, and the bottom of the tissue (plus org) is set to have elevated auxin production. Line 1249 onwards reads:

if(type == C_MINUS)

{

n->import=importInMinus;

n->degradation =minusLevel[0];

}

else if (type== C_PLUS)

{

n->production = plusLevel[0];

}

break;

In the parameter file

PlusLevel: 0.1 0 // Concentration or production rate at the plus side (auxin, PIN)

MinusLevel: 0.01 0// Concentration or degradation rate of the minus side (auxin, PIN)

ImportInMinus: 2

Also, to colour the auxin producing cells at the bottom of the tissue (which is defined in the code as the minus org) orange, and the auxin removal /import cells at the tip blue, the colours of the plus and minus organisers are swapped in the parameter file:

ColorPlus: 80

ColorMinus: 5

**Fig. 16 E and F**

Same as Fig 8A, but an updateOrganisers function is added (line 1714) and called every step (line 1696). This function places the YUC band with elevated auxin synthesis (simulations used in E and F), and the cell with elevated auxin removal and degradation (only in F) into the tissue at time =300.

In E, the part of the updateOrganisers function that positions the cell with elevated auxin removal and degradation is commented out (lines 1727 -1733 and lines 1745-1750).

In the parameter file:

PlusLevel: 0.1 0 // Concentration or production rate at the plus side (auxin, PIN)

MinusLevel: 0.05 0// Concentration or degradation rate of the minus side (auxin, PIN)

ImportInMinus: 0.5

**Fig. 7D**

Based on code used to generate Fig. 8A. In parameter file:

GridSize: 11 13

[Organisers]

Type: None// AuxinProduction CUC None

Plus: none// Left Right Top Bottom CenterLeft CenterRight CenterTop CenterBottom None

Minus: none//

In main code, functions turnIntoSquares (line 579) and findTopAndBottom (line 583) are commented out - so hexagons are not turned into squares and the boundaries are not wrapped (because cells on the boundary are treated like all other cells). In init concentrations (line 1197) function is changed so all cells are initialised with noise in auxin levels:

void initConcentrations()

{

forall const cell& c in T.C:

{

c->auxin = 1 + util::ran(0.05) - 0.05/2;

c->PIN = 0;

c->type=C_NORMAL;

}

forall const junction& j in T.W:

{ forall const junction& jn in T.W.neighbors(j):

{

wall w = T.W.edge(j,jn);

w->PIN = 0;

}

}

}

In the updateDerivatives() function, conditionals testing for whether a cell/ membrane is on the border of the tissue are removed, as is the AuxinFluxAcrossBoundary function.

The PINAllocationToAnEdgeMembrane function is no longer needed so it is removed and the PINAllocationToAMembrane function (line 1770) is simplified as it no longer needs to consider flux across the boundaries.

Drawing of PIN is changed so that its appropriate for hexagons- case WCT_CONCENTRATION on line 2143

**Fig. 7A.**

Based on 7D.

GridSize: 3 3

A new function deleteExtraCells is added- called on line 583 and specified from line 589. This just removes 2 cells to get the arrangement wanted.

Cells and nodes are given a property isCentre (line 107, line 239), this is initially set to be false for all cells / nodes (line 117, line 258). In init concentrations, the central cell has isCentre set to true (line 731). When the solver graph is made, the central cell node also has isCentre set to true (line 825-828). This property is used to govern the reallocation of PIN : PIN binding to the membrane only occurs for the central cell. This is specified on line 1229.

The initConcentrations function is modified so that all cells are initialised with an auxin concentration of 1, and the walls of the central cell are given a noisy PIN concentration.

The auxin production and degradation rates are changed in the parameter file.

AuxinProductionRate:0.25// rho

AuxinTurnover:0.005// mu

The order of updating PIN concentrations and updating auxin concentrations using the solver is changed in the step function, so that auxin concentrations are updated before PIN

**solve(S, *this); //updates auxin concentrations using solver**

dt = solve.dt;

time += dt;

drawTime += dt;

forall const node& n in S:

n->apply(); **// updates the auxin concentrations in the tissue graph**

forall const node& n in S:

{

if (n->type ==NT_MEMBRANE)

{

forall const node& c in S.neighbors(n):

{

if (c->type ==NT_CELL and c->isCentre)

{

n->c[1] =PINallocationToAMembrane(n); // updates the PIN concentrations in

// the membrane

}

}

}

}

In the other simulations it doesn’t make a difference which way round this is done, but here it is important for the auxin concentrations to be updated first. In the alternative case where PIN is updated first, all the outside cells have the same auxin concentration, the noisy initial PIN concentrations will all be set to be the same before there is any effect on auxin concentration.

**Flux based model**

Basic model is Fig. 9A.

**Fig. 9B**

Based on Fig. 9A, but parameters are changed to include elevated auxin import and an elevated rate of degradation at the distal end - the rate of degradation is lower than in 9A.

In the parameter file:

MinusLevel:0.04

ImportInMinus: 30

**Fig. 10A and B.**

Same as Fig. 9B except noise is added to auxin concentrations each step of the simulation after 40 steps, and the updateOrganisers function is run. The only difference to the code is lines 1707-1711 are uncommented. And in the parameter file,

AuxinNoise: 0.5.

**Fig. 16A and B**

Based on 9B. Calling of addNoise function (line 1708) remains commented out (noise is not added to auxin concentrations in this simulations) but updateOrganisers function (line 1712) is uncommented and therefore run. The update organisers function is different to that in the simulation used for Fig. 10A and B. Here this function places a band of cells with elevated auxin biosynthesis (simulations used for 16A and B) and a cell with elevated auxin import and degradation (simulation used for Fig. 16B) when the time of the simulation is > 165 (after the proximo-distal polarity field is established).

Only difference between 16A and B is that in A, lines 1796 to 1803 and lines 1815-1818 are commented out, preventing a cell with elevated auxin import and removal being placed.

**Fig. 7E.**

To make hexgrid, turnIntoSquares and findTopAndBottom are uncommented.

In parameter file:

Organisers: none

Plus: none

Minus: none

Now there is no flux across the boundary, so updateDerivatives function is changed so that the AuxinFluxFromCellToNeighbour function is used to calculate flux across all membranes.

The system is initialised with noise in auxin concentrations. In the initConcentrations function,

Line 1259 is changed to

c->auxin =1 + util::ran(auxinNoise[0]) - auxinNoise[0]/2;

auxinNoise is set to 0.05

Function for drawing PIN is slightly changed- code in WCT_CONCENTRATION

PINWallThickness parameter is changed to 6

Without any changes in parameters compared with Fig. 9A, local regions of tandemly aligned polarities are generated. To get the large scale tandem alignments shown in Fig. 7E, the parameter values were altered:

AuxinProductionRate:0.2// rho

AuxinTurnover:0.005//mu

Alpha: 3.2e-3

Pmax : 0.4

PolarTransport:0.04

**Fig. 7B**

Based on 7E. In parameter file, GridSize: 3 3. On line 594 deleteExtraCells function is called (it just removes 2 cells to get the desired grid).

As for Fig. 7A, cells and nodes given a property isCentre (line 103, line 239), this is initially set to 0 for all cells / nodes (line 114, line 260). In init concentrations, the central cell has isCentre set to true (line 1284). When the solver graph is made, the central cell node also has isCentre set to true (line 1399) and the membrane nodes surrounding the cell also have this property set to true (lines 1456-1463). This isCentre property is used to govern the reallocation of PIN -PIN binding to the membrane only occurs for the central cell. This conditional is specified in update derivatives (line 2083).

The initConcentrations function is modified so that all cells are initialised with an auxin concentration of 1, and the walls of the central cell are given a noisy PIN concentration.

Some parameter values are altered in the parameter file:

AuxinProductionRate: 0.25

Alpha: 4e-3

Coloring: concentration

PINWallThickness: 10

Also, the drawPartitionnedCells1 function is slightly altered so that for small differences in auxin concentration between cells (which occurs before polarisation occurs) cells are coloured correctly.

**Indirect coupling model**

All models are based on that used for Fig. 10C and D.

**Fig 9C.** Same as Fig. 10C and D, but the updateOrganisers function is commented out (line 1715) so the elevated region of auxin biosynthesis in the proximal half of the tissue is not added and cells with elevated auxin import and removal are not induced. Also, unlike in Fig. 10C and D, noise is not added to the auxin concentrations at every step of the simulation, so the addNoise function is commented out (line 1708-1711). Also, the cells at the distal end of the tissue do not have elevated auxin import. This is specified by commenting out line 1074 in the setCellNodeOrganiser function. In the parameter file, the auxin degradation rate at the distal end of the tissue is increased to -7 (MinusLevel: 0 0 -7).

**Fig. 9D.** Same as Fig. 10C and D, but updateOrganisers function is commented out (line 1715) so, as for Fig. 9C, the elevated region of auxin biosynthesis in the proximal half of the tissue is not added and cells with elevated auxin import and removal are not induced. As in Fig. 9D, addNoise is commented out. The behaviour of the top and bottom boundaries are the same as in Fig. 10C and D.

**Fig. 16C and D**

As for Fig. 9D, but now the updateOrganisers function is run. This function is changed compared with the simulation used to generate Fig. 10C and D. Now, when the time is > 65 a band of cells with elevated auxin biosynthesis is added, and in Fig. 16D, a cell with elevated auxin import and removal is also added (only difference between the models is that lines 1779-1788 and 1803-1809 are commented out in Fig. 16C so that the cell with elevated auxin import and removal is not added).

**Fig. 7F**

Based on Fig. 9C.

In parameter file, GridSize : 11 13

Organisers are removed from boundaries in parameter file:

[Organisers]

Type:None//

Plus:None //

Minus:None//

In code, the turnIntoSquares function is commented out so that the hexagonal grid is not turned into staggered squares (line 794).

In the parameter file, the auxin production and degradation rates are changed, as is the rate at which extracellular auxin promotes A* unbinding from the membrane:

Activation: -0.9 0.0 // auxin dependent conversion of A* to A conversion (gamma_aux)

Production:1.3 // production rate of auxin (rho aux)

Degradation:2.0 // degradation rate of auxin (mu aux)

**Fig. 7C**

Based on Fig. 7F. To get the desired grid, in the parameter file, GridSize: 3 3 and in the model code, the function deleteExtraCells is run (as described above for Fig. 7A and 4B). Cells are given the property isCentre (line 115), this is initialised as being false (line 129). In initConcentrations, the central cell is found and has isCentre set to true. Only the central cell is initialised with A, B and PIN in the cytoplasm (line 943-948), other cells do not have A, B or PIN. All cells have the same rates of auxin import and the same initial auxin concentration. The membranes of the central cell initially have noisy A*and B* (line 973), but none of the other cells have A* or B* in the membrane (line 984). Thus, only the central cell has the A and B polarity components and PIN.

In parameter file, Coloring: total_mediator. Draws the auxin concentration in each cell as a shade of green
